# Incorporating a Rapid-Impact Package for Neglected Tropical Diseases with Programs for HIV/AIDS, Tuberculosis, and Malaria: A comprehensive pro-poor health policy and strategy for the developing world

**DOI:** 10.1371/journal.pmed.0030102

**Published:** 2006-01-31

**Authors:** Peter J Hotez, David H Molyneux, Alan Fenwick, Eric Ottesen, Sonia Ehrlich Sachs, Jeffrey D Sachs

## Abstract

Hotez et al. argue that achieving success in the global fight against HIV/AIDS, tuberculosis, and malaria may well require a concurrent attack on the neglected tropical diseases.

*The age of hypocrisy has been succeeded by that of indifference, which is worse, for indifference corrupts and appeases: it kills the spirit before it kills the body. It has been stated before, it bears repeating: the opposite of love is not hate, but indifference. —Elie Wiesel*

The last five years have witnessed increased efforts by G8 nations and United Nations agencies to improve the health of the world's 3 billion people living on less than US$2 a day. Most of this attention has focused on efforts to intensify resources for fighting the three most devastating diseases: HIV/AIDS, tuberculosis, and malaria. Together, these “big three” account for a staggering 5.6 million deaths and the loss of 166 million disability-adjusted life years (DALYs) annually (see annex tables 2 and 3 in [ 1]). Prominent partnerships and initiatives are now devoted to the big three ( Box 1), and increased global attention to these diseases (and to the risks posed by avian influenza and other emerging viral infections) culminated in the November 2005 *TIME* Global Health Summit, branded by Bono as the “Woodstock of Global Health” ( http://www.time.com/time/2005/globalhealth).

These new initiatives and “Woodstock” Global Health have done much to raise funds and elevate public awareness in order to launch a serious war on the big three. Conspicuously absent from these activities, however, has been commensurate advocacy for a group of diseases that exclusively affect the poor and the powerless in rural and impoverished urban areas of developing countries. An increasing body of evidence indicates that this group of “neglected tropical diseases” may not only threaten the health of the poor as much as HIV/AIDS, tuberculosis, or malaria, but even more importantly, may have effective treatment and prevention strategies that can be delivered for less than US$1 per capita per year. Furthermore, new evidence points to substantial geographic overlap between the neglected tropical diseases and the big three, with emerging data suggesting that control of the neglected tropical diseases could actually become a powerful tool for combating HIV/AIDS, tuberculosis, and malaria. Therefore, achieving success in the global fight against HIV/AIDS, tuberculosis, and malaria may well require a concurrent attack on the neglected tropical diseases and waging a larger battle against a new 21st century “gang of four.”

## Global Burden of the Neglected Tropical Diseases

The most important neglected tropical diseases include three vector-borne protozoan infections—leishmaniasis, human African trypanosomiasis, and Chagas disease; three bacterial infections—trachoma, leprosy, and Buruli ulcer; and seven helminth infections—hookworm, ascariasis, trichuriasis, lymphatic filariasis, onchocerciasis, guinea worm (drancunculiasis), and schistosomiasis [ 2]. Cysticercosis, food-borne trematodiases, and some other parasitic infections could also be included in this list [ 3–5]. The common features of the neglected tropical diseases include high endemicity in rural and in impoverished urban areas of low-income countries, and an ability to impair childhood growth, intellectual development, and education, as well as worker productivity. In this way, the neglected tropical diseases are poverty-promoting conditions. Many of the neglected tropical diseases are disfiguring and stigmatizing—their characteristic features are described in the Bible and other ancient texts, affirming that they have affected humans for millennia [ 2]. However, because they affect the poorest of the poor, there are few or no commercial markets for drugs and vaccines against the neglected tropical diseases, and the pharmacopoeia for these diseases has remained essentially unchanged since the middle of the 20th century [ 2].

The burden of disease resulting from the neglected tropical diseases is huge. As shown in Table 1, they cause approximately 534,000 deaths annually, with five diseases—schistosomiasis, hookworm, ascariasis, leishmaniasis, and human African trypanosomiasis—accounting for more than 400,000 deaths. Even more significant than the mortality burden are the years of life lost that result from premature disability. By some estimates, the neglected tropical diseases are second only to HIV/AIDS as a cause of disease burden, resulting in approximately 57 million DALYs annually [ 8]. If considered together, the neglected tropical diseases would represent the fourth most important group of communicable diseases worldwide, behind lower respiratory infections, HIV/AIDS, and diarrheal diseases ( Table 2).

**Table 1:**
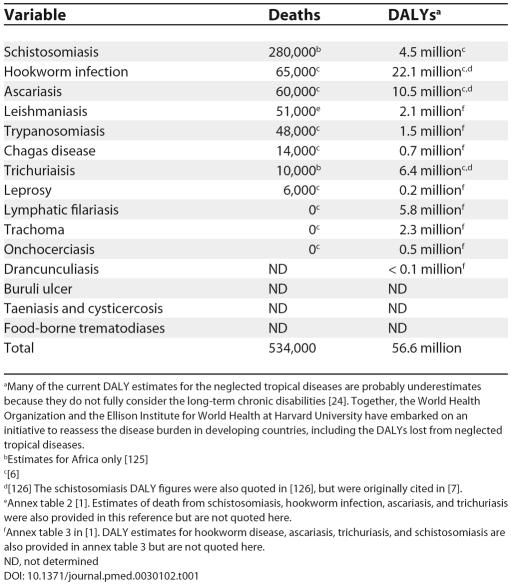
Disease Burden of the Neglected Tropical Diseases in Deaths and DALYs

^a^Many of the current DALY estimates for the neglected tropical diseases are probably underestimates because they do not fully consider the long-term chronic disabilities [ 24]. Together, the World Health Organization and the Ellison Institute for World Health at Harvard University have embarked on an initiative to reassess the disease burden in developing countries, including the DALYs lost from neglected tropical diseases.

^b^Estimates for Africa only [ 125]

^c^[ 6]

^d^[ 126] The schistosomiasis DALY figures were also quoted in [ 126], but were originally cited in [ 7].

^e^Annex table 2 [ 1]. Estimates of death from schistosomiasis, hookworm infection, ascariasis, and trichuriasis were also provided in this reference but are not quoted here.

^f^Annex table 3 in [ 1]. DALY estimates for hookworm disease, ascariasis, trichuriasis, and schistosomiasis are also provided in annex table 3 but are not quoted here.

ND, not determined

**Table 2:**
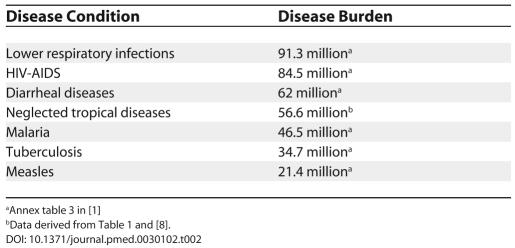
Comparative Disease Burdens of Communicable Diseases Measured in DALYs

^a^Annex table 3 in [ 1]

^b^Data derived from Table 1 and [ 8].

## Epidemiologic Overlap and Comorbidity of the Neglected Tropical Diseases, and Opportunities for Integrated Control

The neglected tropical diseases do not occur in isolation. In most countries of sub-Saharan Africa, and in many other tropical and subtropical countries, at least five to six neglected tropical diseases occur in the same region [ 9]. The implication of this geographic overlap is that a considerable proportion of the population of sub-Saharan Africa (and selected regions of Asia and the Americas) is polyparasitized with one or more soil-transmitted helminths (STHs), schistosomes, and filarial worms [ 10–14], particularly among the very poorest populations [ 15–17]. Each of the three major STHs ( Ascaris lumbricoides, Trichuris trichiura, and the hookworms, Necator americanus and Ancylostoma duodenale) and the two schistosomes ( Schistosoma haematobium and Schistosoma mansoni) are highly endemic to sub-Saharan Africa, where they adversely affect childhood growth and physical fitness [ 18–24]. Such polyparasitism has a substantial impact on the physical health of Africa's youth population [ 25], as well as on the impairment of their intellectual and cognitive development [ 26–30]. Polyparasitism also results in anemia [ 31], with hookworms being well-known causes of anemia because of intestinal blood loss [ 32], though T. trichiura and S. mansoni also cause some degree of intestinal blood loss [ 33–35], and S. haematobium causes anemia through hematuria [ 36]. In addition to host blood loss, the schistosomes also probably cause anemia through other mechanisms including hypersplenism, red blood cell sequestration, autoimmune hemolysis, and chronic inflammation [ 36, 37].

Documented improvements in childhood growth [ 18, 20], physical fitness [ 19, 21], cognition [ 26–30], school attendance [ 38], and hemoglobin and serum ferritin concentrations [ 39–41] following deworming, together with a theoretical framework of helminth transmission dynamics [ 42, 43], provided the basis for a World Health Assembly resolution adopted in 2001 urging member states to periodically deworm school-age children with a benzimidazole anthelmintic (either albendazole or mebendazole) and praziquantel as a means for reducing global disease burden ( http://www.who.int/wormcontrol) [ 44]. Periodic deworming also provides the basic strategy of the Schistosomiasis Control Initiative ( http://www.schisto.org) in Africa [ 13].

Lymphatic filariasis (LF), onchocerciasis, and trachoma exhibit considerable geographic overlap with the STHs and schistosomiasis [ 9]. Therefore, the combination of ivermectin and albendazole for the elimination of LF in Africa and ivermectin for onchocerciasis control—the cornerstones of the Global Programme to Eliminate Lymphatic Filariasis ( http://www.filariasis.org) and the African Programme for Onchocerciasis Control ( http://www.apoc.bf/en), respectively—add considerable benefits to the treatment of ascariasis and trichuriasis [ 45]. At the same time, combinations of ivermectin and azithromycin prevent blindness by reducing the incidence of onchocerciasis and trachoma, respectively [ 46]. Through its impact on ectoparasite infections, ivermectin also indirectly helps to reduce the frequency and severity of skin infections [ 45, 47].

Based on the substantial morbidity reductions afforded by reducing polyparasitism, Molyneux et al. [ 9] have recently put forward a comprehensive pro-poor strategy to integrate programs for either the control or the elimination of seven neglected tropical diseases—ascariasis, trichuriasis, hookworm, lymphatic filariasis, onchocerciasis, schistosomiasis, and trachoma—using existing drugs. Such integration efforts are particularly relevant to sub-Saharan Africa because the neglected tropical diseases in this region exhibit a high degree of geographic overlap [ 9]. It is proposed that such integrated control or elimination could be achieved with four drugs—ivermectin, albendazole, azithromycin, and praziquantel—of which three are currently donated by Merck and Company, GlaxoSmithKline, and Pfizer, respectively, while praziquantel is available at relatively low cost [ 12]. Each of the drugs has overlapping specificity so that multiple neglected pathogens would be concurrently targeted. In addition, the four-drug regimen would also target ectoparasite infections, such as scabies, pediculosis, tungiasis, and cutaneous larva migrans, and their resulting secondary bacterial skin infections [ 45, 47], and possibly also affect important respiratory bacterial pathogens including pneumococcus [ 9]. It has been estimated that for US$200 million annually, approximately 500 million Africans (US$0.40 per patient) could be treated in a four-drug integrated pro-poor package [ 9, 12]. That package could reduce tens of millions of DALYs annually and simultaneously address seven of the eight Millennium Development Goals (MDGs), including those related to poverty reduction, educational achievement, and child and maternal health [ 9]. This approach has been recommended by the Commission for Africa [ 48], and a *Lancet* editorial has emphasized that deworming is a prerequisite for improved health of the world's poorest children and is one of the “quick wins” identified in the Millennium Project Report [ 49, 50].

As scaling up integrated control moves forward, a number of research and monitoring questions will need to be addressed, including issues of compliance [ 51], drug interactions, emerging drug resistance [ 52], and sustainability. Moreover, proof of concept for the feasibility of integrated control will require attention to the specific populations (e.g., children versus adults) at risk for each of the neglected tropical diseases, and to the timing for administration of each of the four drugs. Equally important will be undertaking an economic analysis of the rapid-impact package. The studies on the economic rates of return of large-scale and successful neglected tropical disease control and elimination programs, such as onchocerciasis control in West Africa, guinea worm eradication, LF, and schistosomiasis control in Egypt and China, and Chagas disease control in South America, have shown that these are of the order of 15%–30% [ 53]. The World Bank and other agencies have recognized these programs as being among the most effective development investments in any sector [ 53], and it is hoped that an equal or greater rate of return could be achieved with a rapid-impact package targeting multiple neglected tropical diseases [ 9]. In the future, the concept of integration could be expanded beyond integrated chemotherapy-based morbidity control based largely on community-directed treatment to include access to clean water and improved sanitation; strengthening of surveillance, evaluation, and reporting systems; capacity building, deployment of new generation control tools; and education and communication strategies to address the root ecological and behavioral causes of the neglected tropical diseases (J. Utzinger, personal communication) [ 17, 54, 55].

## Epidemiologic Overlap of the Neglected Tropical Diseases with HIV/AIDS, Tuberculosis, and Malaria

In many parts of the developing world, but especially in sub-Saharan Africa, the geographic overlap between HIV/AIDS, tuberculosis, and malaria is extensive. Indeed, Africa's catastrophic burden of disease resulting from HIV-associated tuberculosis [ 56] and severe malaria in individuals with HIV [ 57–59] is emerging as one of the first great human tragedies of the 21st century. Adding to the complexity of Africa's big three is the geographic and epidemiologic overlay of the neglected tropical diseases (S. Brooker, A. C. A. Clements, P. J. Hotez, S. I. Hay, A. Tatem, et al., unpublished data) [ 60, 61]. It has become increasingly clear that HIV/AIDS, tuberculosis, and malaria occur predominantly in populations who are polyparasitized [ 11, 62, 63]. Helminths are the most common parasites found in HIV-, tuberculosis-, and malaria-infected populations (S. Brooker, A. C. A. Clements, P. J. Hotez, S. I. Hay, A. Tatem, et al., unpublished data) [ 11, 62, 63], especially the three major STHs [ 64–66], the two major schistosomes [ 67–69], and the filariae [ 70]. Brooker and colleagues have recently demonstrated a close geographical overlap in Africa between hookworm and malaria (S. Brooker, A. C. A. Clements, P. J. Hotez, S. I. Hay, A. Tatem, et al., unpublished data). However, almost all of the major neglected tropical diseases have been linked with the big three.

## Comorbidity of the Neglected Tropical Diseases with HIV/AIDS, Tuberculosis, and Malaria: Anemia

We are still in the early stages of appreciating the full extent of the comorbidity that occurs when the neglected tropical diseases are superimposed on HIV/AIDS, tuberculosis, and malaria [ 71]. Anemia has been revealed as perhaps the most important of the leading comorbid conditions. Among the neglected tropical diseases, hookworm, schistosomiasis, kala-azar, and trypanosomiasis are major causes of anemia [ 32, 35, 72, 73], with hookworm accounting for up to 35% and 73% of the iron-deficiency anemia and severe iron-deficiency anemia in Africa, respectively [ 74]. Children and pregnant women are particularly susceptible to anemia from hookworm and schistosomiasis [ 35, 74, 75]. As noted already, hookworm causes iron-deficiency anemia through intestinal blood loss, while the mechanisms of anemia from the other neglected tropical diseases are more complex. Each of the big three also results in anemia, with malaria responsible for the greatest burden. Malaria causes anemia by several different mechanisms, including increased destruction of both parasitized and nonparasitized red blood cells and dyserythropoiesis resulting from host production of inflammatory cytokines, especially tumor necrosis factor and macrophage migration inhibitory factor [ 76, 77]. The pathogenesis of anemia from HIV/AIDS and tuberculosis is also complex, most likely resulting from a state of chronic inflammation leading to bone marrow suppression [ 36].

Coinfection with the neglected tropical diseases also adversely affects the natural history and progression of the big three.

A major consequence of polyparasitism, therefore, is anemia from multiple infectious causes. In the case of hookworm and malaria, Brooker et al. (S. Brooker, A. C. A. Clements, P. J. Hotez, S. I. Hay, A. Tatem, et al., unpublished data) have shown that in Kenya the two types of anemia—anemia from hookworm and the anemia from malaria—can build on each other to produce profound reductions in hemoglobin. To make matters worse, many of these same African populations experience further reductions in hemoglobin because of sickle cell disease and thalassemia, as well as nutritional deficits in iron and folate. Fleming [ 78] put forward the concept of the “agriculture-related anemias” to describe this “perfect storm” confronting African populations—changes in diet, population growth with limited sanitation and with the consequences of endemic STH infections, an environment prone to the emergence of vector-borne disease, and natural selection of variants that offer partial protection against malaria—all of which resulted from the introduction of agriculture, beginning at the time of the Neolithic Revolution.

The severe anemia resulting from helminth polyparasitism and malaria produces several adverse health consequences among three particularly important African subpopulations: pregnant women, children, and individuals with HIV. In pregnancy, anemia is a leading contributor to maternal morbidity and mortality, and is associated with shock, risk of cardiac failure, decreased ability to work, and adverse perinatal outcomes [ 79]. In coastal Kenya, malaria was identified as the most important cause of anemia in primigravidae, whereas hookworm attained increased importance among multigravidae [ 79, 80]. Therefore, women are put at risk by the major consequences of anemia throughout their childbearing years. In young children, chronic iron deficiency and anemia are associated with increased child mortality, and impairments in physical growth, cognitive and motor development, immune function, and school performance [ 81–84]. Severe anemia is also a major contributor to mortality from malaria [ 85]. Just as women are at risk for anemia throughout their childbearing years, first from malaria and then from hookworm, so, too, are children whose first major experience with severe anemia is from malaria during early childhood, followed by hookworm and schistosomiasis in middle childhood and adolescence. Together, these infectious anemias account for an important component of the “silent burden of anemia” in African children [ 86]. Finally, among individuals with HIV, anemia has been shown to be an independent risk factor for early death, with correction of anemia associated with reversal of increased risk [ 87, 88].

## Impact of the Neglected Tropical Diseases on Host Susceptibility to HIV/AIDS, Tuberculosis, and Malaria

Coinfection with the neglected tropical diseases also adversely affects the natural history and progression of the big three. Several studies (recently reviewed in [ 62]) point to the increasing severity of clinical malaria that results from helminth coinfection. These studies include those from Senegal showing enhanced risk or increased incidence of clinical malaria resulting from either STH [ 64, 66] or schistosome [ 68] infections, unpublished studies from Malawi showing that women infected with hookworms were at 1.8 times higher risk of having malaria than uninfected women [ 89], and studies from Thailand showing increased susceptibility to malaria in patients with STH infections [ 90–93].

However, not all studies investigating these relationships have identified deleterious interactions between helminths and malaria [ 63, 69, 94, 95]. Indeed, there may be a difference between helminthic effects on susceptibility to malaria and effects on the pathology induced by malaria infection. Druilhe et al. [ 62] have hypothesized that the STHs and schistosomes immunomodulate the host to increase malaria susceptibility by shifting their humoral responses from malaria-protective, cytophilic humoral antibodies (IgG1 and IgG3) to nonprotective, noncytophilic subclasses (IgG2, IgG4, and IgM). In addition, Diallo et al. [ 67] have proposed that schistosomes, and possibly other helminths, can unbalance the regulation of inflammatory cytokines in childhood malaria.

Another body of evidence links helminth coinfections with increased susceptibility to HIV/AIDS or worsening progression of HIV disease (reviewed in [ 65]). Borkow and Bentwich postulate that chronic helminth infections increase the risk of HIV infection through a process of chronic immune activation [ 96], which results in increased HIV plasma viral load [ 97–99]. Among a group of Kenyan car washers with high levels of exposure to S. mansoni infection, Secor et al. [ 100] have shown that patients with active schistosomiasis exhibit increased expression of the chemokine receptors and HIV-1 co-receptors, CCR5 and CXCR4, on peripheral blood CD4 T cells and monocytes. Gallagher et al. [ 101] observed increased risk of mother-to-child HIV transmission in pregnant women with helminth coinfection; increased risk correlated with cord blood lymphocyte production of interleukin-5 and interleukin-13 in response to helminth antigens. However, studies conducted in Uganda failed to identify an association between helminths and AIDS [ 102, 103]. In addition to promoting susceptibility to HIV/AIDS, one major neglected tropical disease in Africa, namely, visceral leishmaniasis, is an important opportunistic infection in individuals who are HIV-immunocompromised [ 72].

Finally, there is some evidence that helminth infections, especially hookworm and schistosomiasis, adversely affect the outcome of pulmonary tuberculosis or the progression to active tuberculosis [ 104, 105], and reduce the T cell responses in individuals receiving Bacillus Calmette–Gurerin (BCG) [ 106–108]. However, the data supporting this concept is still far from conclusive.

Taken together, this evidence indicates that coinfection with one or more neglected tropical disease may profoundly affect the outcome of one or more of the big three. Progression of disease from HIV/AIDS, tuberculosis, and malaria results from the comorbidity associated with anemia from hookworm, trichuriasis, schistosomiasis, trypanosomiasis, and leishmaniasis, and from the possible increase in susceptibility and worsening progression of disease that occurs with STH, schistosome, and filarial infections. However, the latter concept is still not without controversy and requires additional scientific investigations.

## A Comprehensive Pro-Poor Health Policy

It is important to determine if there is a rationale for linking a pro-poor strategy for integrated neglected tropical disease control with ongoing programs that target the big three. Recent evidence of the extensive geographic overlap between the big three and the neglected tropical diseases, together with the deleterious interactions between both groups of infections, suggests significant, new opportunities to reduce the burden of disease in sub-Saharan Africa and elsewhere in the developing world. Success at integrating neglected tropical disease control into big three partnership programs could dramatically reduce the number of life years lost from premature death and disability in Africa [ 9]. The collateral benefits from including neglected tropical disease control under the umbrella of the HIV/AIDS, tuberculosis, and malaria global partnerships are potentially huge.

As noted above, integrated control of the neglected tropical diseases relies on the appropriate use of three anthelmintics—albendazole, ivermectin, and praziquantel—and the antibacterial agent azithromycin. In addition to affecting STH and schistosome deworming, ectoparasite control, and control or elimination of LF and onchocerciasis, the three anthelmintics would help to reduce anemia as well as the progression of disease resulting from HIV/AIDS, tuberculosis, and malaria [ 62, 65]. In Senegal, deworming for STH infections was shown to be equivalent to the protection from malaria conferred by the sickle cell trait [ 62, 64], and has the added effect of restoring T cell responses to mycobacterial antigens in helminth-exposed individuals before and after BCG vaccination [ 107, 108]. In Zimbabwe, treatment for schistosomiasis reduced the rate of HIV-1 viral replication and increased CD4 cell counts among individuals who were coinfected [ 99], although this phenomenon was not observed in Ugandans who were coinfected [ 109]. Therefore, it may be important to consider local and geographical differences when evaluating the impact of deworming on coinfections.

In addition to the beneficial effects of deworming on children already mentioned, antenatal anthelmintic treatment was shown recently to reduce maternal morbidity and mortality, and improve birth weight and infant survival [ 110]. The community-based health-care systems set up to deliver neglected tropical disease drugs could be well suited to administer antiretrovirals, directly observed tuberculosis therapy, and antimalarials and bed nets. Simultaneously, neglected disease control would benefit from big three control efforts. For example, insecticide-treated bed nets used in malaria control are highly effective at interrupting the transmission of LF [ 70], and many antiretrovirals would be expected to reduce the impact of opportunistic leishmaniasis.

New vaccines under development for HIV/AIDS, tuberculosis, and malaria also need to account for the influence of polyparasitism on vaccine immunogenicity [ 111]. In laboratory animals, the presence of adult hookworms in the intestine reduces vaccine-specific antibody titer and lymphoproliferation, with restoration occurring following deworming [ 112], and S. mansoni infection reduces the protective efficacy of BCG vaccination [ 113]. Studies in humans also show similar effects of concurrent helminth infections on immune responses to tetanus and cholera vaccines [ 114, 115]. However, a recently published study found that although maternal hookworm infection was associated with reduced maternal immune response to mycobacterial antigen, such maternal infection was also associated with an unexpected increase in their infants' response to BCG [ 116].

It is surprising that those aiming to control the big three have largely ignored these opportunities.

Since any vaccine trial that does not control for worm infections could be flawed, neglected tropical disease control should be linked with big three vaccine development efforts, such as those sponsored by the International AIDS Vaccine Initiative ( http://www.iavi.org), the Malaria Vaccine Initiative ( http://www.malariavaccine.org), and the Aeras Global Tuberculosis Vaccine Foundation ( http://www.aeras.org). Deworming prior to vaccination may also become a key component in the testing process of any new generation vaccine against the big three. Moreover, development of vaccines against the neglected tropical diseases themselves, including vaccines for hookworm [ 117], schistosomiasis [ 118], and leishmaniasis [ 119] is also underway. In the future, the neglected tropical disease vaccines are expected to become important control tools; in the case of hookworm and schistosomiasis vaccines, their deployment would reduce the frequency of chemotherapy and, therefore, possibly reduce the likelihood of emerging anthelmintic drug resistance [ 120].

Given the compelling logic and the very modest costs of embracing neglected tropical disease control efforts, it is surprising that those aiming to control the big three have largely ignored these opportunities and the collateral benefits from reductions in anemia, worm burdens, and susceptibility to HIV/AIDS, tuberculosis, and malaria morbidity. Bilateral donors and the major big three partnerships should expand their portfolios to incorporate deworming, LF, and onchocerciasis elimination efforts, and other neglected tropical disease control initiatives as a cost-effective means to reduce the morbidity and mortality of HIV/AIDS, tuberculosis, and malaria. Such interventions are inexpensive, effective, and fully compatible with the MDGs, the recommendations of the UN Millennium Project ( http://www.unmillenniumproject.org), the Commission for Africa report [ 48], and a Resolution of the Third Global Meeting of the World Health Organization Partners for Parasite Control [ 44]. While the recent report on health from the New Partnership for Africa's Development ( http://www.nepad.org), an initiative to develop an integrated socioeconomic development framework for Africa with a mandate from the Organisation of African Unity [ 121], and the policy statement on health from the European Parliament [ 122] refer to parasitic diseases, they do not emphasize the interrelationships between parasitic infections and HIV/AIDS, tuberculosis, and malaria, or the potential benefits of a more holistic approach to disease control. The excellent safety profile of the anthelmintics and azithromycin, and the fact that three of the four drugs are donated free of charge to those who need them, would almost certainly ensure that neglected tropical disease control could be incorporated without added risk and with minimal costs.

## Scaling Up Disease Control

The international community has started to address issues of scaling up disease control in the developing world. HIV/AIDS, tuberculosis, and malaria have been given a financing mechanism for scale up through the establishment of the Global Fund to Fight AIDS, Tuberculosis, and Malaria. The scaling up of malaria control was explicitly featured in the final outcome document of the September 2005 UN World Summit ( http://www.un.org/ga/59/hlpm_rev.2.pdf). The World Bank and other donors are similarly identifying new channels and financing strategies for infectious disease control [ 123].

The scale up of control measures vis-à-vis the neglected tropical diseases can be readily added to these ongoing initiatives, possibly by first incorporating neglected tropical disease control into model health systems. Two great examples are the Tanzania Essential Health Interventions Project—a research and development partnership between Tanzania's Ministry of Health and Canada's International Development Research Centre ( http://www.idrc.ca)—and the Millennium Village Project, a new development project guided by a scientific council at the Earth Institute at Columbia University ( http://www.earth.columbia.edu/mvp) and based on the recommendations of the UN Millennium Project. The Millennium Village Project will provide a framework for scaling up control of the neglected tropical diseases alongside other health interventions in project sites in ten African countries, with rigorous surveying and monitoring of the MDG-based indicators. These issues will be subjects of important discussions at a January 2006 summit in Stockholm, Sweden, as both programs recognize that it is the countries themselves who must eventually define appropriate policy and priority, often through a decentralized system of district-level teams. Attending to the neglected tropical diseases will require committed support for baseline mapping of the prevalence of the individual neglected tropical diseases, scientifically vetted treatment protocols, and appropriate alignment of these efforts with regional and national health policies.

## Conclusion

The neglected tropical diseases have joined the ranks of the big three to create a 21st century “gang of four.” For too long the big three partnerships have worked in isolation, as have the vertical programs against the individual neglected tropical diseases. The recent Paris Declaration emanating from the High-Level Forum on the Health MDGs calls for greater harmonization and collaboration across the major global health partnerships and the commended efforts by the smaller neglected tropical disease partnerships (the minor global health partnerships) to integrate these activities [ 124]. In the future, policy must be driven by the reality of the biological and epidemiological interactions between the big three and the neglected tropical diseases. Coordination of the major and minor global health partnerships and their associated research communities would give a significant thrust to the efforts now underway to reduce disease and poverty worldwide.

**Box 1. Prominent Partnerships and Initiatives Devoted to the “Big Three”**

- **The Joint UN Programme on HIV/AIDS** ( http://www.unaids.org)
- **Roll Back Malaria** ( http://www.rollbackmalaria.org)
- **Stop Tuberculosis** ( http://www.stoptb.org)
- **The Global Fund to Fight AIDS, Tuberculosis, and Malaria** (So far has attracted US$4.7 billion in financing through 2008 [ http://www.theglobalfund.org])
- **The US Leadership against HIV/AIDS, Tuberculosis, and Malaria Act of 2003** (An emergency effort to provide US$15 billion over five years [ http://olpa.od.nih.gov/actions/public/108session1/pl108-25.asp]),
- **The President's Emergency Plan for AIDS Relief** ( http://www.avert.org/pepfar.htm),
- **The Bill and Melinda Gates Foundation** ( http://www.gatesfoundation.org), supporting the development of new technologies for disease control.

## Abbreviations

BCG: Bacillus Calmette–Gurerin
DALY: disability-adjusted life year
LF: lymphatic filariasis
MDG: Millennium Development Goal
STH: soil-transmitted helminth
