# Correction: Incorporating a Rapid-Impact Package for Neglected Tropical Diseases with Programs for HIV/AIDS, Tuberculosis, and Malaria

**DOI:** 10.1371/journal.pmed.0040277

**Published:** 2007-09-25

**Authors:** Peter J Hotez, David H Molyneux, Alan Fenwick, Eric Ottesen, Sonia Ehrlich Sachs, Jeffrey D Sachs

Correction for:

Hotez PJ, Molyneux DH, Fenwick A, Ottesen E, Ehrlich Sachs S, et al. (2006) Incorporating a Rapid-Impact Package for Neglected Tropical Diseases with Programs for HIV/AIDS, Tuberculosis, and Malaria. PLoS Med 3(5): e102. doi:10.1371/journal.pmed.0030102

In Table 1, under the column labeled “Deaths,” there should be an “e” footnote for the deaths from trypanosomiasis, Chagas disease, and leprosy, not a “c” as written.

